# New Appliances and Things Medical

**Published:** 1894-03-17

**Authors:** 


### NEW APPLIANCES AND THINGS MEDICAL.

[All preparations, appliances, novelties, &c., of which a notice is desired, should be sent for the Editor, to care of The Manager, 428, Strand, London, W.C.]

### TABLOIDS OF ALUMNOL, AGATHIN, BENZOSOL, IODOPYRIN, DERMATOL, AND HYPNAL.

(Burroughs and Wellcome, Snow Hill, E.C.)

We have been supplied with samples of the above new drugs in a new or compressed form. Some of them have been pretty extensively used in England, others have yet to make a reputation for themselves. Physicians in England will do well to keep pace with the march of modern therapeutics, and if they cannot initiate, at least follow in the footsteps of their more enterprising brethren on the Continent, for it is unnecessary to state that these drugs, if not exclusively made, are at least invented in Germany.

*Alumnol* is a new astringent and antiseptic, and is the more interesting since it is the outcome of a synthetic process, and manufactured on rational lines for a set purpose, and not a therapeutic agent of accidental discovery. It is one of the many drugs which may be said to be the product of the "new therapeutics." It is a compound of aluminium in combination with naphtol sulphuric acids, and its chief virtue lies in its non-coagulability with albuminous discharges, hence when applied to the secreting surface of a wound it is able to permeate the deeper strata of the tissues and there hasten the processes of repair in virtue of its astringent and antiseptic properties. It is chiefly recommended for indolent chronic ulcers, endometritis gonorrhœica, and various chronic skin affections.

*Agathin* (salicyl alpha methyl phenyl hydrazone) is an analgesic of reputed reliable properties; it appears to be peculiarly valuable in cases of intermittent neuralgia, sciatica, and rheumatic conditions.

*Benzosol* (benzoyl guaiacol) is a preparation which is intended to take the place of creosote in the treatment of phthisis; it is free from the objectionable taste and after effects of the latter drug. In the system it becomes decomposed into benzoic acid and guaiacol; the latter can be detected in the sweat and urine within a few hours of ingestion, hence its antiseptic properties can exercise a prompt action on the foci of infection in the lungs. In consequence of this property it has been regarded as a specific for pulmonary consumption.

*Iodopyrin*, is antipyrin in which one atom of hydrogen is replaced by one of iodine (iod phenyl demethyl pyrazolon). Within the stomach it is supposed to become decomposed into antipyrin and sodium iodide, so that the combined antipyritic properties of the two drugs have a double effect. It is recommended for the pyrexia of typhoid and tuberculosis.

*Dermatol* is a basic gallate of bismuth; it has astringent and drying properties, it may be applied to external wounds and areas of eczema, or internally for gastric ulcers and intestinal catarrh. It is intended as a vulnary to replace iodoform. It cannot be said that at present it has in any way done so. It deserves, however, a more extensive trial.

*Hypnal* is a combination of chloral hydrate and antipyrin. It is, perhaps, better known to the profession in England than any of the above-mentioned drugs. We have used it ourselves somewhat extensively in hospital practice, and as a hypnotic it has much to be said for it in preference to chloral and sulphonal. It is pleasant to take, and has no disagreeable after effect.

Messrs. B. and W. are doing a distinct service to medicine in introducing these drugs in so handy a form as that afforded by their compressed tabloids.

### PURE MALVERN SPRING WATER—THE "ALPHA" BRAND.

(W. and T. Burrows, Malvern.)

The purity of the Malvern waters is not only a matter of tradition, it is a solid fact. And to obtain these waters many patients have been recommended to visit Malvern. Now, thanks to Messrs. Burrows, the waters of Malvern can be delivered at one's door, and if kept in a cold cellar, are just as refreshing and agreeable as when drunk on the spot. At the present day, with the huge demand for aerated and mineral waters, the conditions of competition have opened the way to many abuses in the manufacture and sale of such waters. It is useful, therefore, for the public as well as the profession to be familiar with the name of a good brand on which they can rely. The "Alpha" brand of mineral waters is one which we are prepared to recommend. The manufacturer attaches great importance to the facilities he offers for the obtaining of the pure unaerated water. Those who like a still water, cannot do better than drink the "Alpha" brand. It also has advantages in invalid cooking and the mixing of medicines. For our part we much prefer the simple aerated water, which one may describe as a brilliantly sparkling water, and one absolutely free from organic impurity. This water lends itself most happily to admixture with spirits, and for this purpose has many advantages over the soda, lithia, and potash waters, all of which have their advantages in other respects, and for the treatment of gout and rheumatism they are waters on which reliance may safely be placed. Those desirous of making trial of the Malvern waters may find an advantage in doing so before visiting the town. If a taste in London proves satisfactory, it is pretty certain that the drinking of them on the spot will fully justify a visit.

